# A Proteomic Approach to Study the Biological Role of Hepatitis C Virus Protein Core+1/ARFP

**DOI:** 10.3390/v14081694

**Published:** 2022-07-31

**Authors:** Vasileios Vrazas, Savvina Moustafa, Manousos Makridakis, Ioannis Karakasiliotis, Antonia Vlahou, Penelope Mavromara, Katerina R. Katsani

**Affiliations:** 1 Laboratory of Biochemistry and Molecular Virology, Department of Molecular Biology and Genetics, Democritus University of Thrace, 68100 Alexandroupolis, Greece; vasivraz1@mbg.duth.gr (V.V.); pmavrom@mbg.duth.gr (P.M.); 2 Clinical Immunology-Rheumatology Unit, 2nd Department of Medicine and Laboratory, Hippokration General Hospital of Athens, 11527 Athens, Greece; savinabio@hotmail.com; 3 Centre of Basic Research, Biomedical Research Foundation of the Academy of Athens, 11527 Athens, Greece; vlahoua@bioacademy.gr (A.V.); mmakrid@bioacademy.gr (M.M.); 4 Laboratory of Biology, Department of Medicine, Democritus University of Thrace, 68100 Alexandroupolis, Greece; ioakarak@med.duth.gr

**Keywords:** proteomics, HCV-1a, Core+1/ARFP, Huh7.5, liver diseases

## Abstract

Hepatitis C virus is the major cause of chronic liver diseases and the only cytoplasmic RNA virus known to be oncogenic in humans. The viral genome gives rise to ten mature proteins and to additional proteins, which are the products of alternative translation initiation mechanisms. A protein—known as ARFP (alternative reading frame protein) or Core+1 protein—is synthesized by an open reading frame overlapping the HCV Core coding region in the (+1) frame of genotype 1a. Almost 20 years after its discovery, we still know little of the biological role of the ARFP/Core+1 protein. Here, our differential proteomic analysis of stable hepatoma cell lines expressing the Core+1/Long isoform of HCV-1a relates the expression of the Core+1/Long isoform with the progression of the pathology of HCV liver disease to cancer.

## 1. Introduction

Hepatitis C virus (HCV) is an enveloped RNA virus of the Flaviviridae family, transmitted through contaminated blood [1]. HCV targets primarily human liver cells, evades innate and adaptive immunity, and establishes chronic infections in 70% of cases. Ultimately, infection by HCV leads to hepatocellular carcinoma (HCC) in 20% of the cases [2], a unique feature for a flavivirus [3,4]. The virus circulates in the blood plasma associated with low-density lipoproteins, LDLs, and very-low-density lipoproteins VLDLs and enters liver cells via clathrin endocytosis [5]. Replication of HCV takes place in the cytoplasm at specialized membrane compartments derived from the ER and induced by the virus [6]. The viral genome is a 9.6-kb single-stranded positive-sense RNA that encodes a polyprotein precursor of approximately 3000 amino acids, that is processed into at least ten mature structural and non-structural proteins (Core, E1, E2, p7, NS2, NS3, NS4A, NS4B, NS5A, and NS5B) by host and viral proteases [7]. 

The viral genome is then packaged into a capsid composed of the Core protein (C), covered by a lipid bilayer embedding multiple copies of the two structural glycoproteins The viral life is concluded by the release of the viral particles through the secretory pathway without cell lysis [8]. HCV morphogenesis and propagation rely on lipid metabolism [9,10]. Host cells have evolved mechanisms of detecting viral infection and then altering the host translation capacity to restrict viral protein production. However, viruses, especially RNA viruses such as HCV, are repositories of functional RNA elements that can exploit the host cell’s translation machinery, even before viral proteins accumulate [11]. Interestingly, just as other viruses such as picornaviruses have done, HCV has evolved alternative mechanisms to initiate translation and circumvent host defense mechanisms [12]. Moreover, many positive-sense RNA viruses, which infect mammalian cells, present Alternative Reading Frame Proteins (ARFPs) in their genomes, which are also translated. The existence of such ARFPs poses some questions concerning their role during the infection since they do not directly affect the viral replication in the cell-based infection systems but may contribute to virus replication in vivo, during natural infection, thus representing virulent factors [13]. One such ARFP is an alternative open reading frame (ORF) overlapping the Core coding region in the +1 frame of genotype 1 and synthesizing another viral protein by a ribosomal frameshift mechanism within an A-rich area. The protein was named ARFP, F, or Core+1 protein [14,15,16,17]. The ARFP is conserved among the different HCV genotypes [18]. However, in the case that this repetitive A-rich sequence is absent, as in most HCV isolates of genotype 1, no frameshift is detected. Interestingly, in transfected cells, the predominating isoforms of Core+1/ARFP (genotype 1a) are generated by internal translation initiation at codons 85/87 (Core+1/Short) [19] or codon 26 (Core+1/Long) [20]. Conserved RNA stem-loops (SL) SL47 and SL87 of the HCV Core-encoding region are important for viral genome translation in cell culture and in vivo as well as for mediating the internal translation initiation of the alternative Core+1/Short ORF [21]. The expression of the two isoforms was also demonstrated in hepatoma Huh7-Lunet cells transfected with replicons of the JFH1 HCV isolate (genotype 2a) [22]. The detection of Core+1/ARFP-specific antibodies and T-cell responses in HCV-infected patients have also indicated that the isoforms are also expressed in vivo [23,24,25,26]. 

The biological significance of these alternative translation isoforms is still under investigation. Experiments in chimpanzees (HCV genotype 1a), mice (HCV genotype 1a/JFH1), and culture cells (HCV genotype 2a) implied that Core+1/ARFP is not required in replication [21,27], a fact embedded in the study of Core+1/ARFP. On the other hand, several independent studies provided preliminary evidence for the contribution of Core+1 protein in advanced liver disease and liver cancer (refs in [18,28]). The presence of specific natural occurring mutations within the Core/Core+1 region in clinical samples isolated from liver biopsies of HCC patients demonstrated that a different distribution of Core+1/ARFP quasi-species between tumoral and non-tumoral liver tissue may occur [29]. Another important role of Core+1/ARFP that emerged from several studies is the modulation of the immune system. Core+1/ARFP can modulate dendritic cell function leading to stimulation of T cells [30]. Moreover, studies on an in vitro model system based on isolated human immune cells infected with recombinant adenovirus vectors carrying the Core/Core+1 sequences reported that Core+1/ARFP induces several cytokines involved in hepatic injury [31]. Notably, expression of Core+1/ARFP seems to relate to decreased IFNα production by peripheral blood mononuclear cells (PBMC) in hepatitis C patients [32], while it attenuates type I and type III IFN responses to RIG-I/MDA5 PAMPs in Huh7 cells [33]. Consistent with this finding, a positive correlation between the prevalence of Core+1/ARFP antibodies and lack of response to the standard IFN /RBV therapy was recently shown [34]. Nonetheless, additional work is required to clarify the mechanisms whereby Core+1/ARFP interferes with innate immune responses. Proteomics has been used extensively to identify cellular pathways perturbated by viral proteins and virus biology [35,36]. 

To address the function of the Core+1/ARFP isoforms we have established stable Huh7.5 cell lines expressing the Core+1/Short and Core+1/Long isoforms of the HCV genotype 1a [37]. To further address the functional role of the Core+1/ARFP isoforms we performed proteomic analysis on the stable Huh7.5 Core+1/ARFP expressing cell lines and the annotated differentially expressed proteins were used in functional pathways analysis.

## 2. Materials and Methods

### 2.1. Cell Lines

The Huh-7.5 cell lines expressing the Core+1/Long and Core+1/Short isoforms of HCV-1a and the control Gun (empty vector) were described in [37]. Cells were cultured in Dulbecco’s modified Eagle’s medium supplemented with nonessential amino acid, 2 mM L-glutamine (Gibco™, Waltham, MA, USA), 100 ug/mL penicillin–streptomycin (Gibco™), and 10% fetal bovine serum at 37 °C and 5% CO_2_ with the addition of G418 (1 mg/mL). Cells were treated with 2 mM hydroxyurea for 24 h before analysis. 

### 2.2. Sample Preparation for MS Analysis

Samples were homogenized in FASP lysis buffer (4% SDS, 0.1 M DTE, 0.1 M Tris-HCl pH 7.6). Protein concentration was determined by the Bradford assay. Protease inhibitors (Roche, Basel, Switzerland) were added at a final concentration of 3.6% and samples were stored at −80 °C until further use. Protein extracts (200 μg/sample) were processed using filter aided sample preparation (FASP) as described previously [38], with minor modifications [39]. Briefly, buffer exchange was performed in Amicon Ultra Centrifugal filter devices (0.5 mL, 30 kDa MWCO; Merck, Kenilworth, NJ, USA) at 14,000 rcf for 15 min at room temperature. The protein extract was mixed with urea buffer (8 M urea in 0.1 M Tris-HCl pH 8.5) and centrifuged. The concentrate was diluted with urea buffer and centrifugation was repeated. Alkylation of proteins was performed with 0.05 M iodoacetamide in urea buffer for 20 min in the dark followed by centrifugation at 14,000 rcf for 10 min at RT. Additional series of washes were conducted with urea buffer (two times) and ammonium bicarbonate buffer (50 mM NH_4_HCO_3_ pH 8.5, two times). Tryptic digestion was performed overnight at RT in the dark, using a trypsin to protein ratio of 1:100. Peptides were eluted by centrifugation at 14,000 rcf for 10 min, lyophilized, and stored at −80 °C until further use.

### 2.3. LC-MS/MS Analysis

All LC-MS/MS (liquid chromatography-tandem mass spectrometry) experiments were performed on the Dionex Ultimate 3000 UHPLC system coupled with the high-resolution nano-ESI Orbitrap-Elite mass spectrometer (Thermo Scientific, Waltham, MA, USA). Each sample was reconstituted in 200 μL loading solution composed of 0.1% *v*/*v* formic acid. A 5 μL volume was injected and loaded on the Acclaim PepMap 100 (100 μm × 2 cm C18, 5 μm, 100Ȧ) trapping column with the ul PickUp Injection mode with the loading pump operating at a flow rate of 5 μL/min. For the peptide separation the Acclaim PepMap RSLC, 75 μm × 50 cm, nanoViper, C18, 2 μm, 100Ȧ column retrofitted to a PicoTip emitter was used for multi-step gradient elution. Mobile phase (A) was composed of 0.1% formic acid and mobile phase (B) was composed of 100% acetonitrile, 0.1% formic acid. The peptides were eluted under a 240-minute gradient from 2% to 80% (B). Flow rate was 300 nL/min and column temperature was set at 35 °C. Gaseous phase transition of the separated peptides was achieved with positive ion electrospray ionization applying a voltage of 2.5 kV. For every MS survey scan, the top 10 most abundant multiply charged precursor ions between *m*/*z* ratio 300 and 2200 and intensity threshold 500 counts were selected with Fourier Transform (FT) mass resolution of 60,000 and subjected to HCD fragmentation. Tandem mass spectra were acquired with FT resolution of 15,000. Normalized collision energy was set to 33 and already targeted precursors were dynamically excluded for further isolation and activation for 30 s with 5 ppm mass tolerance.

### 2.4. MS Data Processing and Quantification

Raw files were analyzed with Proteome Discoverer 1.4 software package (Thermo Finnigan, San Jose, CA, USA), using the Sequest search engine and the Uniprot human (Homo sapiens) reviewed database, including 20,204 entries. The search was performed using carbamidomethylation of cysteine as static and oxidation of methionine as dynamic modifications. Two missed cleavage sites, a precursor mass tolerance of 10 ppm, and fragment mass tolerance of 0.05 Da were allowed. False discovery rate (FDR) validation was based on q value: target FDR: 0.01.

### 2.5. MS Data Analysis

The Student’s two-tailed t-test was performed on the normalized values of the Long (L), Short (S) and Vector (V) groups. Τhe distributions of the expression levels of L versus V replicates (and S versus V replicates) were evaluated with F-test for each gene to examine whether they are homoscedastic or heteroscedastic. If the value of the F-test was less than or equal to 0.05 the distributions were considered heteroscedastic for the *t*-test, else if the value of the F-test ranged between 1 and 0.05, the distributions were considered homoscedastic [40]. Subsequently, a two-tailed *t*-test was applied with homoscedastic or heteroscedastic distribution assumption derived from the F-test to the gene expression levels of V and L. The average value of the three biological replicates was calculated and the ratio between the L group and V group for each protein of the group gave the relative fold change of the protein expression, differentially expressed proteins (DEPs) in the L group were selected the ones with a *p*-value of *t*-test ≤ 0.05, had at least three peptides assigned by the mass analyzer, and their expression was ≥2-fold or ≤0.5-fold different from the control (V group proteins) [41].

### 2.6. Functional Network Enrichment Analysis

The DEPs were used as input for STRINGDB to create a functional network [42]. The sources used for the analysis and the creation of the network were text-mining, experimental data, gene fusion data, co-reference in databases, co-expression data, common cell topological neighborhood, and co-occurrence. The network was enriched with fifty-first protein neighbors of the DEPs, obtained from the same sources. Non-experimental sources were also used. The threshold was set at 0.7 for each one of the fifty entries to be considered statistically significant and included in the network.

### 2.7. GSEA Analysis 

Gene set enrichment analysis is based on Smirnoff–Kolmogorov test, which calculates the probability of finding out the number of genes belonging to the same pathway randomly [43]. Gene set enrichment analysis was performed using the Broad Institute software (http://software.broadinstitute.org/gsea/, accessed on 1 July 2022). Accordingly, selected DEPs were ranked according to their negative decimal logarithm of the *p*-value and assigned a negative value to them if they were down-regulated. The “Hallmark database 7.4” used for the analysis and the resulted ranks were then inputted into a Preranked Gene Set Analysis, using the following parameters: at least three genes needed to consider a hallmark enriched, the metric used was signal-to-noise ratio, the enrichment was weighed, the normalization mode was meandiv. The results of the analysis were then evaluated and only hits with FDR values ≤ 0.27 were accepted. Statistical significance was calculated using 1000 permutations.

### 2.8. Bibliographic Searches

All the bibliographic searches were performed using the DisGeNet database [44].

### 2.9. Visualisation of Proteomics Data

Both Heatmap, PCA, as well as the mapping of genes on KEGG pathways, were generated with R (https://www.R-project.org/, accessed on 1 July 2022) using the dplyr, stringr, gplots, pathview, gage, and gageData packages. 

### 2.10. Cellular Pathway Analysis Using KEGG Annotations

KEGG pathways analysis was performed via R-GAGE enrichment analysis against the pathways of KEGG [45] with FDR threshold < 0.25 and downloading the pathways that had at least 2 DEPs featured.

### 2.11. RNA Isolation, cDNA Synthesis, and qPCR 

Total RNA was isolated from cells according to the NucleoZOL protocol (Magerey–Nagel, Düren, Germany) was used to synthesize cDNA using the Takara Smart MMLV protocol according to the manufacturer’s instructions with an additional final phenol: chloroform: isoamylic alcohol purification step. qPCR was performed on an ABI7500 real-time PCR system (Applied Biosystems, Waltham, MA, USA). Transcripts were detected using the following sets of primers: EPB41L2 (5′-GAGCTGCACAAA-ACCCACAG-3′, 5′-CCAGCTTGATGTCCACACCT-3′), CTTN (5′-AGGCCGACCGAGTAGACAA-3′, 5′-TATTTGCCGCCGAAACCT-3′), ACTN1 (5′-GGGGACACAGATCGAGAA-3′, 5′-TGTGCACTCTCATCTTGCC-3′), HRAS (5′-CCCTTGGGTGTCAAAGGTAAA-3′, 5′-AAACTGATGCGTGAAGTGCTG-3′), Ykt6 (5′-TCAGCGTCCTCTACAAAGGC-3′, 5′-AGCGCTCCACAATCAGTTG-3′).

### 2.12. Wound-Healing Assay

Wound-healing assay was performed using an Ibidi wound healing kit (Cat. No. 81176) according to the manufacturer s guidelines. Images were captured at different time points on an inverted microscope using 40× magnification and then analyzed with ImageJ [46]. The calculation of cell migration velocity was done using the following calculations [47]:

$$t_{1/2gap}=\frac{InitialGapArea}{2|slope|}\;v_{migration}=\frac{|slope|}{2\times l}$$

where *t*_1/2*gap*_ is the time it takes for the gap to close to half the original area, and *v_migration_* is the cell migration rate.

## 3. Results

### 3.1. Proteomic Analysis of the Huh 7.5/ARFP Expressing Cell Lines

**Figure 1:**
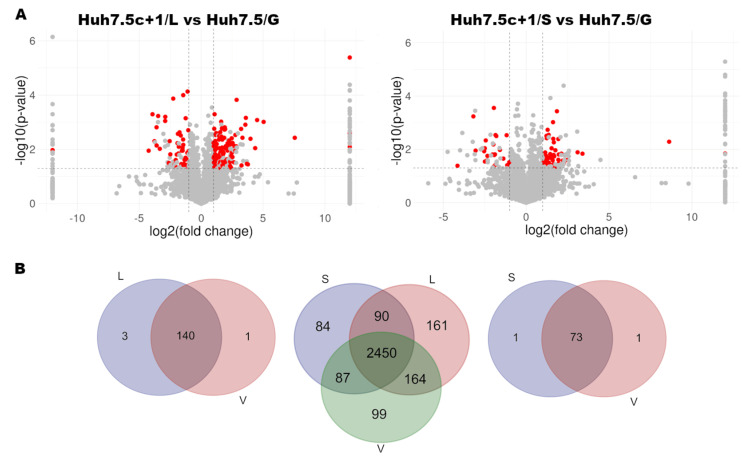
(**A**) Volcano plots of the normalized L (left) and S (right) data sets. (**B**) Venn diagrams showing the overlap of all samples (center), of L versus control V filtered mass spec data (left), of S versus control V filtered mass spec data (right).

To identify the cellular proteins induced by the expression of the HCV Core+1/ARFP by a proteomic-based approach, lysates were prepared from the established Huh7.5 cell lines that constitutively express the Core+1/ARFP (Long and Short isoforms) [37]. Three independent biological replicates constituted of two individual clones and the initial pool of cellular clones were analyzed. Cell lysates were trypsin-digested and analyzed by liquid chromatography-tandem mass spectrometry (LC-MS/MS). Of the 2866 proteins identified in the L sample compared to 2755 in the control (V) sample, 144 were considered differentially expressed proteins (DEP) with a ≥2- or ≤0.5-fold difference from the control and at the same time *p* < 0.05 (Supplementary File S2). Moreover, they were identified in all three replicates (frequency 3) and had at least three unique peptides detected. Of the 144 DEPs in the L sample, 103 proteins were up-regulated, whereas 41 proteins were down-regulated. Only three proteins, TMP4, CADM1 and RTCA, were specific to the Huh7.5c+1/L cell line and not detected in the control Huh7.5/G cell line (Figure 1A). 

Similarly, we analyzed data from the Huh7.5c+1/S cell line stably expressing the Core+1/Short isoform. The normalized protein list included 2711 proteins of the S sample. After applying the same filters as for L, 74 proteins were defined as the DEPs set of the S sample (Figure 1B, Supplementary File S2). Notably, the mass analyzer did not register any Core+1/Long and or Core+1/Short peptides (Supplementary File S1). However, their expression was confirmed by immunofluorescence and western blot, provided the cells were treated with the proteasome inhibitor MG132, otherwise they are both unstable proteins, especially the Short form [37].

### 3.2. The Expression of Core+1/ARFP Changes the Proteome of the Huh7.5 Cells

**Figure 2:**
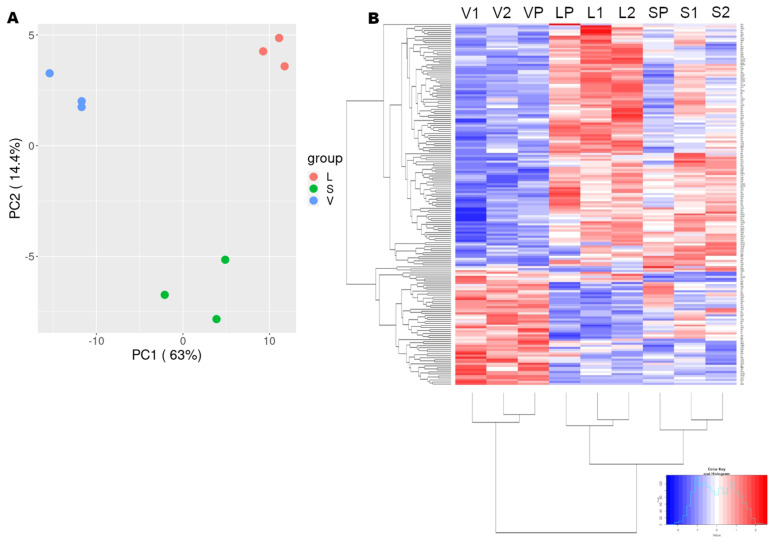
(**A**) PCA of the filtered mass spectrometry data. (**B**) Heatmap of the filtered mass spectrometry data. V1, V2, VP, the biological replicates of the Control V sample. L1, L2, LP, the biological replicates of the L sample. S1, S2, SP, biological replicates of the S sample.

Principal component analysis (PCA) and the heatmap of the normalized raw mass spectrometry data showed that the cell lines used in the analysis clustered in three distinct groups that differ from each other quite significantly (Figure S1). Of note, after filtering, using the three set criteria: first, log2(fold-change) > +/−1 (>2 or <0.5), second, the *t*-test between the sample and control replicates should be (−log10(*p*-value)) > 1.3, and third, the mass analyzer identified at least three unique peptides per protein, the positions of the individual replicates on the PCA graph did not change drastically, but their relative distances did change, bringing the replicates of each group closer to each other. Also, the variance described by the PC1 increased substantially, in comparison to the PCA graph of the unfiltered data, from 28.8% to 63% (Figure 2A). The filtered heatmap of the DEPs demonstrated an obvious pattern of gene expression. The genes were split into two large clusters that were down-regulated in the V group, while up-regulated in L and S and vice versa (Figure 2B). The unfiltered mass-spectrometry data were also clustered together. There was only a small cluster in the S group which is down-regulated and in V, while up-regulated in L. 

Conclusively, the mass spectrometry measurements were reproducible within one group of biological replicates and the changes in the expression levels caused by the expression of Core+1/Long or Core+1/Short isoforms pointed to the same direction.

### 3.3. Functional Network Enrichment Analysis Using the STRINGDB

**Figure 3:**
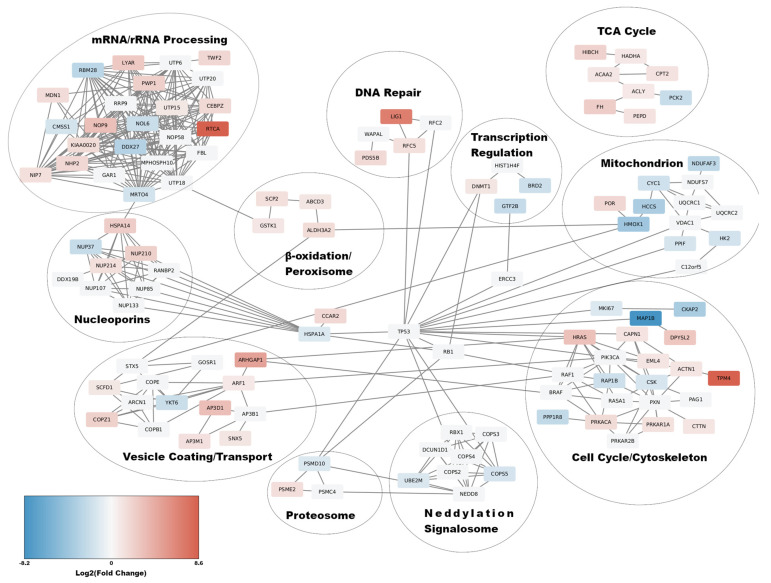
Functional Network Enrichment to the DEPs of the L sample by STRINGDB. The differentially expressed proteins include only high confidence entries (significance > 0.7). Red boxes: up-regulated proteins; blue boxes: down-regulated proteins; white boxes: first neighbors. Scale bar, log2 (fold-change).

To discover the impact of the Core+1/Long protein expression on the cell functions, we applied Functional Network Enrichment analysis to the DEP data set of L. Therefore, the 144 DEPs were used as input in the STRING database (STRINGDB), in order to create a functional human protein network [42]. For the creation of the network, all types of first neighbors were used and the confidence threshold for each of them was set to mildly strong (significance ≥ 0.7). The resulting functional network depicted a sub-proteome that was directly affected by the expression of Core+1/Long with a resulting *p* = 5.39 × 10^−11^. Enrichment of the network with 50 first neighbors resulted in a network highly interconnected between the members and a low probability of emersion of such network by chance (*p*-value = 2.33 × 10^−15^). The functional network was clustered into eleven functional cluster (Figure 3): mRNA/rRNA Processing, Nucleoporins, Vesicle Coating/Transport, Neddylation/Signalosome, Proteosome, β-oxidation/Peroxisome, Cell cycle/Cytoskeleton, Mitochondrion, TCA Cycle, Transcription Regulation, and DNA Repair (Figure 3). More specifically, in the mRNA/rRNA processing group, all genes related to rRNA processing, ribosome assembly, as well as various ribosome subunits were up-regulated, apart from CMSS1 and NOL6, which were down-regulated. On the other hand, proteins related to mRNA processing, RBM28, DDX27, and MRTO4, were down-regulated apart from CEBPZ, which is up-regulated and possibly affects the upregulation of heat-shock proteins [48]. The transcription factors GTF2B and BRD2 from the transcription regulation cluster were found down-regulated as well (Figure 3). Three differentially expressed nuclear pore proteins appeared in the network, that belong to different nuclear pore compartments, NUP37, which is a member of the Y-complex and is downregulated, NUP214, an FG-nucleoporin associated with the nucleocytoplasmic transport of proteins, and NUP210, a transmembrane nucleoporin (Figure 3). The expression of Core+1/Long seems also to affect cellular metabolism. All genes in the mitochondrion cluster, such as HCCS, NDUFAF3, and CYC1, except for POR, that are involved in the energy production through oxidative phosphorylation are down-regulated, while genes involved in lipid metabolism and β-oxidation and connected to TCA cycle, such as CPT2, HADHA, ACAA2, and ACLY were up-regulated. HK2 from the mitochondrion cluster and PCK2 from the cluster TCA cycle cluster, which are predominantly involved in glycolysis were also down-regulated (Figure 3). The majority of proteins that constitute the cytoskeleton cluster in the network were up-regulated. In particular, HRAS, ACTN1, TPM4, CTTN, DPYSL2, and RAP1B are involved in the organization of the cytoskeleton and the development of actin filaments (Figure 3). All these proteins were up-regulated, except for RAP1B. There were also proteins related to microtubules and mitotic spindle development, such as MAP1B, which is downregulated, and PRKACA, EML4, and PRKAR1A, which were up-regulated. Finally, LIG1, RFC5, and PDS5B, which are involved in DNA damage repair, were also up-regulated (Figure 3). All proteins located in the Vesicle Coating/Transport cluster, except YKT6, were up-regulated (Figure 3). PSME2 and PSMD10 are members of the proteasome. PSME2, which acts in the proteolysis of peptides before they are transported to the MHCI proteins, is up-regulated. On the other hand, PSMD10 is involved in the negative regulation of tumor suppressors RB1, and p53/TP53 is down-regulated [49]. Finally, two proteins, UBE2M and COPS5, present in the Neddylation/Signalosome cluster, were down-regulated. Additionally, the differentially expressed proteins were mapped in similar KEGG pathways such as tight junction (hsa04530), cytoskeleton (hsa04810), MAPK Signaling (hsa04010), antigen processing and presentation (hsa04612), endocytosis (hsa04144), fatty acid degradation (hsa00071), and peroxisome (hsa04146) (Table 1).

The corresponding functional network of Core+1/Short was dismissed as non-significant (*p*-value: ~0.2) (Supplementary File S3). 

### 3.4. Functional Analysis Using Gene Set Enrichment Analysis (GSEA)

**Figure 4:**
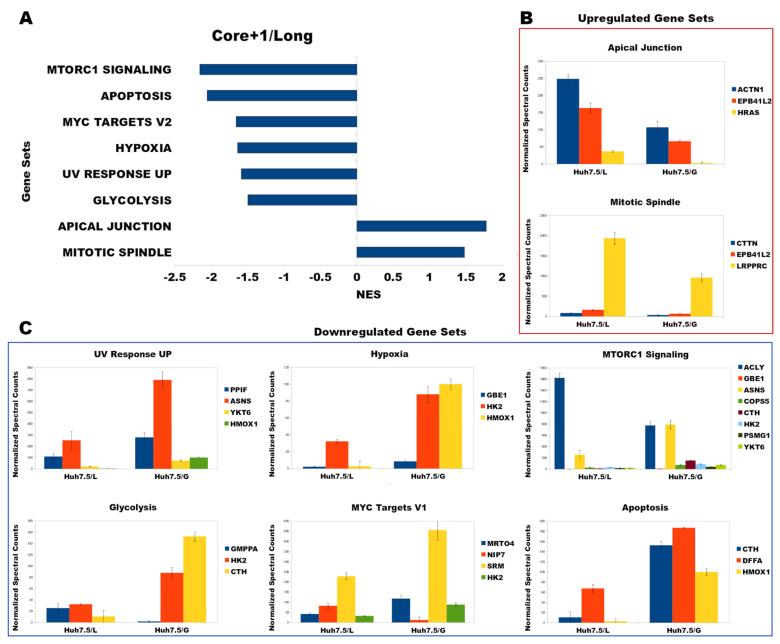
(**A**) GSEA of the 144 DEPs identified hallmarks in sample L (FDR ≤ 0.27, and nominal *p* < 0.05). (**B**) Upregulated enriched sets and the normalized protein levels found in sample. (**C**) Downregulated enriched sets and the normalized protein levels found in sample L. GSEA, gene set enrichment analysis. NES, normalized enrichment score. FDR, false discovery rate.

In our attempt to discover the significant cell functions and pathways that are modified directly by the expression of Core+1/Long, we performed Gene Set Enrichment Analysis (GSEA), in conjunction with the Functional Network Enrichment Analysis. The 144 DEPs of the L sample were used to generate a Gene Rank list (see in Materials & Methods). The enrichment analysis was performed against the Molecular Signatures Database (MSigDB) hallmark gene set collection to reduce the redundancy and noise, because of the analysis. The criteria set was that gene sets had to include at least three members and their false discovery rate (FDR) had to be below or equal to 0.27 (FDR ≤ 0.27). According to the above set criteria, the GSEA of the 144 significant proteins revealed that they fall mainly into eight functional categories (Table 2), and most of them were characterized as cancer hallmarks. The “up-regulated” gene sets were related to the formation of the mitotic spindle and to apical junction (Figure 4A), while the down-regulated functional “hallmarks” were the: MTORC1 pathway, apoptosis signaling pathways, MYC targets, hypoxia, response to UV radiation, and glycolysis (Figure 4B). Notably, the functional network enrichment analysis of the S data did not yield any meaningful results (Supplementary File S4). 

### 3.5. Correlation of GSEA Results with Liver Pathology

Next, the genes from the enriched pathways were searched against the DisGeNET database for possible correlations with liver pathologies. Interestingly, the regulation of the HRAS, CTTN, YKT6, DFFA, ACTN1, EPB41L2, ASNS, HMOX1, and PPIF proteins in L could be correlated with that of HCV-infected hepatocytes. In particular, the up-regulation of GTPase HRAS is associated with carcinoma [50], while up-regulation of Cortactin and down-regulation of YKT6 and DFFA are related to tumor cell invasiveness [51,52,53] and down-regulation of PPIF/CypD may lead to steatosis [54]. Finally, up-regulation of alpha-actinin-1, Band 4.1-like protein 2 (EPB41L2), asparagine synthetase (ASNS), and down-regulation of heme oxygenase (HMOX1) are correlated with cirrhosis [55] (Table 3). Conclusively, misexpression of HRAS, CTTN, YKT6, DFFA, ACTN1, EPB41L2, ASNS, HMOX1, and PPIF is related to advanced liver diseases frequently observed in patients infected with HCV, indicating that Core+1/Long may play an important role in the development of liver pathology. After the selection of the most significant genes using GSEA and the DisGeNET database, we decided to test the expression levels of HRAS, CTTN, ACTN1, EPB41L2, and YKT6, which are involved in carcinoma (HRAS), tumor invasion (CTTN, YKT6), and cirrhosis (EPB41L2, ACTN1) and cross-validate them with the mass spectrometry results. The mRNA levels were 1.97-fold for EPB41L2, 1.43-fold for ACTN1, 1.47-fold for CTTN, 1.72-fold for HRAS, and almost invariable 1.19-fold for YKT6, while their protein levels were almost two-fold up-regulated, with the exception of YKT6, which was found to be down-regulated (Supplementary File S2). 

### 3.6. Core+1/Long Stable Expression in Huh7.5 Cells Increases Cell Migration

**Figure 5:**
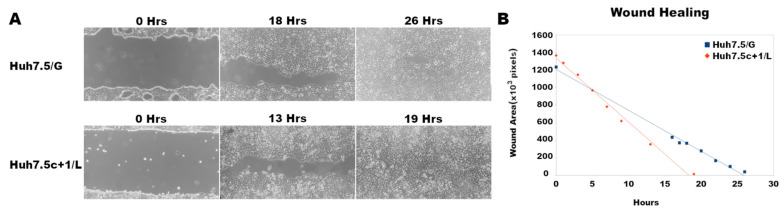
The expression of Core+1/Long increases the migration of Huh7.5c+1/L cells. (**A**) Representative images of a wound-healing assay in Huh7.5/G (control) and Huh7.5c+1/L cell monolayers. (**B**) Quantitation of the wound closure over 30 h of cell migration.

Differential expression of CTTN and of YKT6 has been related to cell migration and tumor invasion [51,53]. This led us to the hypothesis that the expression of Core+1/Long in Huh7.5 cells may transform them into highly invasive tumor cells. To test the occurrence of this phenotype, cell migration velocities were calculated for Huh7.5c+1/L and control cells in a wound-healing assay. We observed the cell migration for 26 h for the Huh7.5/G cell line and for 19 h for the Huh7.5c+1/L cell line (Figure 5A). The cell migration rate was extrapolated from the slope of the plot of the gap area as a function of time (Figure 5B, Supplementary File S5). In summary, the Huh7.5c+1/L cell line closed its wound much faster than the Huh7.5/G. As shown in Figure 5, the wound-healing rate, and by extension, the cell migration velocity (72,840 pixels/h), in Huh7.5c+1/L is approximately 1.56 times faster than in the control (46,577 pixels/h), as shown by the slopes of the plot.

## 4. Discussion

Here, we used a proteomic approach to shed light on the functional role of HCV Core+1/ARFP protein via analyzing mass spectrometry data of Huh7.5 cells stably expressing it. Differentially expressed proteins (DEPs) with relative fold change ≥2 or ≤0.5 and *t*-test *p*-value ≤ 0.05 were further used in functional pathway analysis.

As reported before, the expression of Core+1 isoforms (Core+1/Long and Core+1/Short) in stably transfected Huh7.5 cells accelerated cell cycle progression and increased mRNA levels of proliferation-related oncogenes. Moreover, the Core+1/Short isoform was found before to enhance liver regeneration and oncogenesis in transgenic mice [37]. Increased proliferation was associated with the induction of cyclin D1 expression and Rb phosphorylation. It was also discovered that the p21 CDK inhibitor was enhanced in the presence of both Core+1/ARFP isoforms, possibly promoting the oncogenic form of p21 [57]. Finally, serological tests displayed a high prevalence of anti-Core+1/ARFP antibodies in patients with HCV-induced HCC or advanced cirrhosis compared to control groups, suggesting a link of Core+1/ARFP with virus pathogenesis in the late stages of infection [23,25]. Our findings after the analysis of the mass spectrometry data concerning Core+1/Long also supports these findings. Notably, we found via GSEA that the enriched hallmarks consisted of genes such as HRAS, DFFA, CTTN, YKT6, PPIF, ACTN1, EPB41L2, HMOX, and ASNS are responsible for liver pathology phenotypes, namely as cirrhosis, steatosis, oncogenesis, and tumor invasion (Table 3).

This fact adds evidence to the hypothesis that Core+1/ARFP may be contributing to the development of HCC and cirrhosis by altering the expression profiles of these genes. The non-precise correlation of protein levels with transcripts levels of the selected genes we observed may be attributed to differences in transcription and translation regulation [58] and differences in the biological replicates used. 

According to the STRINGDB clustering (Figure 3), the expression of Core+1/Long protein reduces the expression of genes involved in mRNA processing and transcription, while enhancing the expression of genes involved in ribosome assembly and rRNA processing. These findings could indicate that Core+1/Long may play a role in hijacking the host cell translation machinery during HCV infection. Since the processing of mRNA is hindered, cellular mRNA export from the nucleus may be inhibited, and by extension, cellular mRNA translation may be limited. As a result, the cytoplasmic (+) ssRNA genome of HCV is translated in higher amounts by the increased number of ribosomes, whose function is enhanced by Core+1/Long. Moreover, the blockage of expression of selected innate immunity genes, even indirectly, may be another mechanism used by the virus to manipulate the host [59]. It should be noted that an experimental system mimicking HCV pathogenesis is still lacking; therefore, all experiments were conducted on an immortalized cancer cell line with altered homeostasis compared to normal hepatocytes, which is an over-simplification of an organ with its intricate multi-cell type structure lacking the contribution of the immune system. 

Many RNA and most DNA viruses manipulate nucleocytoplasmic transport and its components [60,61]. Despite the fact that it is a cytoplasmic replicating virus, HCV exploits the nuclear transport machinery to establish a favorable environment for its replication [62]. Our proteomic analysis revealed three nuclear pore proteins affected by Core+1/Long expression. Nup37 has a positive role in the progression of HCC and other cancers as well [63], while its silencing induces inhibition of cell proliferation, G1 phase cell cycle arrest, and apoptosis in non-small cell lung cancer cells [64]. Surprisingly, NUP37 was found to be down-regulated in the L sample, possibly due to the hydroxyurea treatment that arrested cells in the G1/S phase. NUP214 is an FG -nucleoporin that participates in the shuttling of NF-κB and latent STAT1 into the nucleus [65]. Interestingly, NUP214 together with transmembrane nucleoporin NUP210 is one of the nucleoporins subverted by dsDNA viruses, (+) ssRNA viruses such as coronaviruses, and ssRNA-RT viruses such as HIV-1 [61,66]. NUP210 expression was one of the host genes affected during equine encephalitis virus (VEEV) infection [67] and was overexpressed during primate infection by the Zaire Ebolavirus (ZEBOV) mRNA [68] and in adenovirus-infected cells [69], but was down-regulated by Dengue Virus Type 2 in a cell-based assay [70]. 

We also observed that Core+1/Long may cause metabolic reprogramming of the host cell. The expression of Core+1/Long in Huh7.5 cells caused the downregulation of genes related to oxidative phosphorylation, while simultaneously up-regulating genes related to β-oxidation. These alterations usually cause oxidative stress to cells, which was also observed during infection by HCV [71,72]. Metabolic and oxidative stress could be easily attributed to protein overexpression, however, Core+1/ARFPs are very unstable proteins and not highly expressed in the Huh7.5 stable cell lines [73]. 

The role of a COPI protein complex in the HCV infection life cycle that relies on its involvement in vesicle trafficking is not fully defined. Interestingly, the COP1Z subunit of the COPI complex identified in our analysis was also identified in a functional genomic screen for viral replication genes [74], while a physical interaction between an HCV protein and a subunit of the COPI protein complex has also been reported [75]. Core+1/Long may be associated with the construction of virions in endoplasmic reticulum (ER) since HCV lipoviroparticles assemble in the ER and bud off from it to the Golgi compartment in COPII vesicles [76]. The discovery of up-regulated genes related to vesicle transport could lead to the assumption that Core+1/Long may play a role in virus replication; however, previous in vivo studies did not support this notion [27], [77]. Remarkably, the tumor suppressor protein p53 is a central hub that regulates all the affected pathways. Of note, both the HCV Core protein and the Core ARFP have been shown to deregulate the p53 pathway [78,79], while it has been suggested that mutations in the HCV genome Core region are associated with increased HCC risk [28].

We also observed that genes associated with cytoskeleton and focal adhesion, such as ACTN1 and CTTN, are up-regulated and are related to HCV-mediated cirrhosis [80]. In addition, the up-regulation of these and other proteins associated with the cytoskeleton and cell cycle progression, such as HRAS, may explain the fast growth of the Huh7.5 cells expressing Core+1/ARFP. Our wound-healing assay proved the predicted phenotype that cells expressing Core+1/ARFP migrate faster than control cells. Remarkably, the Huh7.5 cells expressing Core+1/Long displayed significantly higher plate adhesion in comparison to the control cells, a fact which may also be attributed to the over-expression of genes associated with the cytoskeleton, and apical junction. 

Psme, a protein linked to the proteolysis of peptides that are transported to the MHCI, was also found up-regulated [49]. Moreover, the molecular chaperone protein HSPA1A or Hsp70 is down-regulated (Figure 3). Hsp70 is responsible for the transport of peptides to the MHCI [81]. Therefore, we may hypothesize that HCV infected cells activate pathways for antigen presentation via MHCI and the expression of Core+1/Long hinders this pathway by preventing Hsp70 to transport the proteolyzed peptides to MHCI, thus modulating the immune response. Okamoto et al. have related HCV RNA replication with FKBP8 and Hsp90, and their interaction with NS5B was reported as well [75,82]. 

In summary, while a correlation between HCC and the expression of the Core+1/ARFP isoforms has been speculated in the past, here we provide the first molecular signature that the existence of Core+1/ARFP may trigger oncogenesis and metastasis. 

## Figures and Tables

**Table 1:** KEGG pathways related to the DEPs of sample L and the associated genes. Green: down-regulated, red: up-regulated. KEGG: Kyoto Encyclopedia of Genes and Genomes.

KEGG PATHWAY (Pathway ID)	Gene Names				
Tight junction (hsa04530)	CTTN	ACTN1	PKA		
Actin Cytoskeleton (hsa04810)	ACTN1	HRAS	-	-	-
MAPK Signaling (hsa04010)	HRAS	PKA	RAP1	HSP72	-
Antigen processing and Presentation (hsa04612)	PA28	HSP70	-	-	-
Endocytosis(hsa04144)	ARF	HRAS	HSC70	SNX5	-
Fatty acid degradation (hsa00071)	MFP	HADHA	ACAA	CTP2	ALDH
Peroxisome(hsa04146)	SPX	ABCD	GSTK1	PMP70	-

**Table 2:** Enriched pathways using the 144 DEPs of L.

Regulation	Hallmarks	Enriched Genes	Protein Names
**Up** **regulated**	APICAL JUNCTION	ACTN1	Alpha-actinin-1
		EPB41L2	Band 4.1-like protein 2
		HRAS	GTPase Hras
		
	MITOTIC SPINDLE	CTTN	Src substrate cortactin
		EPB41L2	Band 4.1-like protein 2
		LRPPRC	Leucine-rich PPR motif-containing protein
			
**Down-regulated**	MTORC1 SIGNALING	ACLY	ATP-citrate synthase
		GBE1	1,4-alpha-glucan-branching enzyme
		ASNS	Asparagine synthetase
		COPS5	COP9 signalosome complex subunit 5
		CTH	Cystathionine gamma-lyase
		HK2	Hexokinase-2
		PSMG1	Proteasome assembly chaperone 1
		YKT6	Synaptobrevin homolog YKT6
		
	APOPTOSIS	CTH	Cystathionine gamma-lyase
		DFFA	DNA fragmentation factor subunit alpha
		HMOX1	Heme oxygenase 1
		
	MYC TARGETS V1	MRTO4	mRNA turnover protein 4 homolog
		NIP7	60S ribosome subunit biogenesis protein homolog
		SRM	Pyrimidine/purine nucleoside phosphorylase
		HK2	Hexokinase-2
		
	HYPOXIA	GBE1	1,4-alpha-glucan-branching enzyme
		HK2	Hexokinase-2
		HMOX1	Heme oxygenase 1
		
	UV RESPONSE UP	PPIF	Peptidyl-prolyl cis-trans isomerase F
		ASNS	Asparagine synthetase
		YKT6	Synaptobrevin homolog YKT6
		HMOX1	Heme oxygenase 1
		
	GLYCOLYSIS	GMPPA	Mannose-1-phosphate guanyltransferase alpha
		HK2	Hexokinase-2
		CTH	Cystathionine gamma-lyase

**Table 3:** Genes associated with liver pathology according to the DisGeNET database [44].

Gene Symbol	Phenotype	References
**HRas**	Carcinoma	[50]
**CTTN**	Tumor cell invasion	[53]
**DFFA**	Tumor cell invasion	[52]
**YKT6**	Tumor cell invasion	[51]
**PPIF**	Steatosis	[54]
**ACTN1**	Cirrhosis	[55]
**EPB41L2**	Cirrhosis	[55]
**HMOX**	Cirrhosis	[56]
**ASNS**	Cirrhosis	[55]

## Data Availability

Not applicable.

## Supplementary Materials

The following supporting information can be downloaded at: https://www.mdpi.com/article/10.3390/v14081694/s1, Figure S1: (A) PCA of the normalized raw mass spectrometry data. (B) Heatmap of the normalized raw mass spectrometry data V1, V2, VP, the biological replicates of the Control group V. L1, L2, LP, the biological replicates of the cell line expressing Core+1/Long (L). S1, S2, SP, the biological replicates of the cell line expressing Core+1/Short (S); Figure S2: mRNA levels of five genes related to liver pathology in the Huh7.5c+1/L cell line. ** *p* < 0.01, ns, non-significant; File S1: Raw data. The full list of the cellular proteins identified by MS; File S2: DEPs; File S3: STRINGDB clusters from Figure 3 and associated genes; File S4: List of GSEA enriched pathways, Enrichment-Core+1-L, and Enrichment-Core+1-S; File S5: wound healing assay.
