# Supplementary figures and images for: A Proteomic Approach to Study the Biological Role of Hepatitis C Virus Protein Core+1/ARFP
Source: Viruses. 2022 Jul 31;14(8):1694. doi: 10.3390/v14081694 (PMC9518822; doi:10.3390/v14081694)

# Fold Change

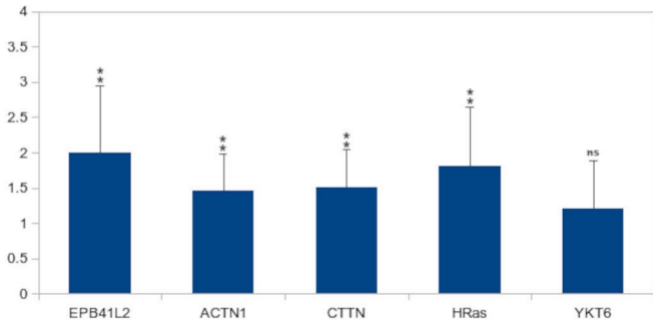

Supplement: Supplementary file 1 [file viruses-14-01694-s001.zip › Supplementary_Figure_S2.pdf]

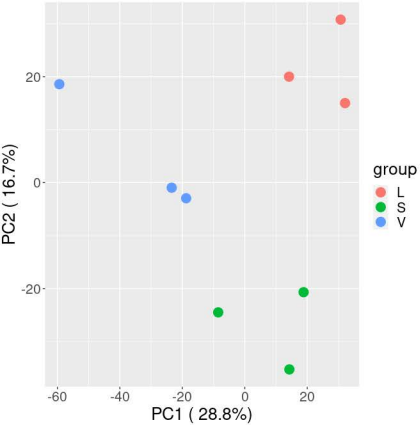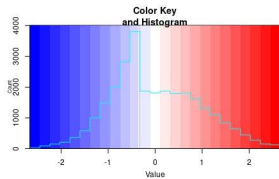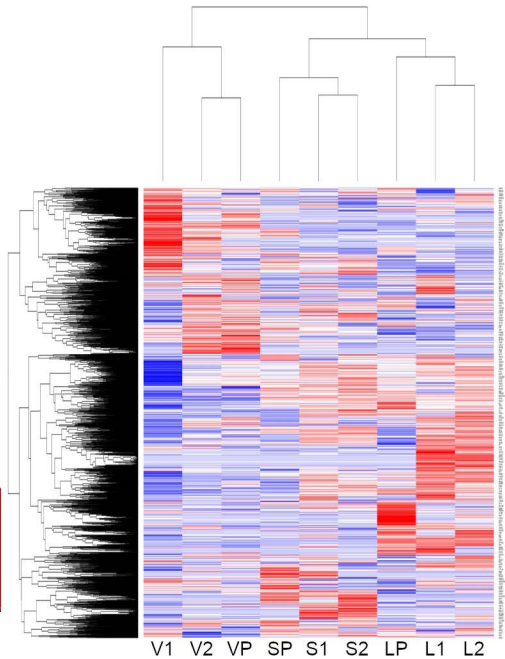

Supplement: Supplementary file 1 [file viruses-14-01694-s001.zip › Supplementay_Figure_S1.pdf]
